# Light-regulated root hair development? HY5 is a coordinator

**DOI:** 10.1093/plphys/kiae344

**Published:** 2024-06-26

**Authors:** Yee-Shan Ku

**Affiliations:** Assistant Features Editor, Plant Physiology, American Society of Plant Biologists; School of Life Sciences and Centre for Soybean Research of the State Key Laboratory of Agrobiotechnology, The Chinese University of Hong Kong, Hong Kong SAR, China

Root hair enlarges root surface to facilitate processes such as nutrient uptake and plant-microbe interactions. Root hair development is regulated by various hormones. For example, auxin is known to promote root hair initiation and elongation (Vissenberg et al. 2020). However, the full picture of the signaling network involved in root hair development is far from complete.

**Figure 1.**
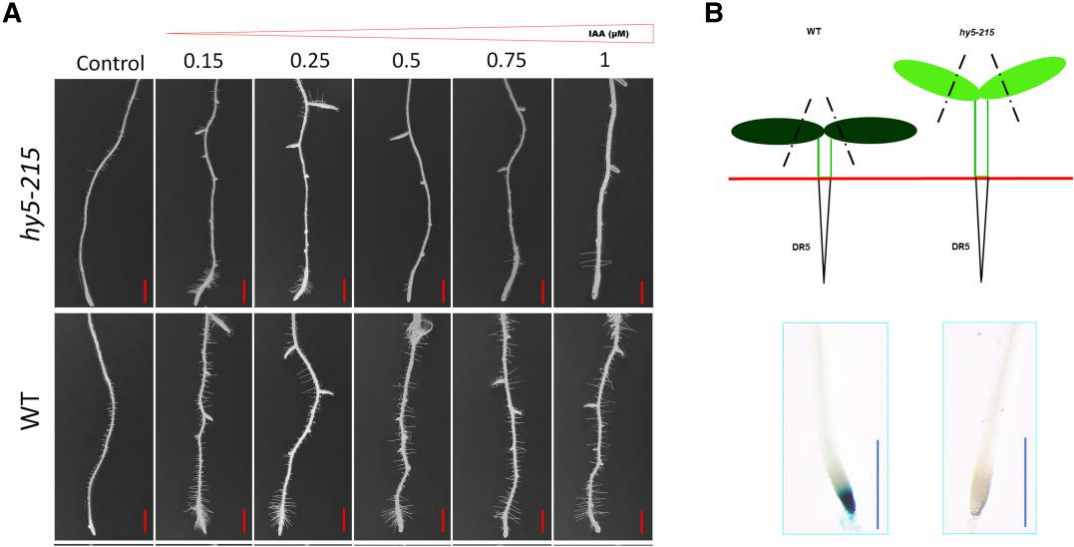
Shoot-to-root signaling regulates root hair development. **A)** IAA treatment promoted root hair density in wild-type (WT) plants. The *hy5-215 mutant* reduced the number of root hairs. The hy5 root hair defects could be partially rescued by externally supplied IAA at low concentrations. **B)** Root stock expressing *GUS* driven by auxin-responsive *DR5* promoter was grafted with different scions. Compared with wild-type scion, *hy5* mutant scion led to the decreased auxin level at the root tip (Fig. 1B). The figure is modified from Gaddam et al. (2024).

In this issue of *Plant Physiology*, Gaddam et al. reported a root hair growth regulatory network comprising of a bZIP transcription factor HY5 (ELONGATED HYPOCOTYL 5), the microRNA397b, and arabinosylation-related genes in Arabidopsis (Gaddam et al. 2024). Disruption of *HY5* reduced root hair density, and the phenotype could be partially rescued by externally supplied auxin indole-3-acetic acid (IAA) at low concentrations (Fig. 1A). The *hy5* mutants also had lower expression of genes for auxin signaling, transport, and biosynthesis. HY5 is a shoot-to-root mobile transcription factor that coordinates light-activated root growth (Chen et al. 2016). To further determine whether the mobile transcriptional factor HY5 regulates root hair development by modulating auxin level in the root, the authors analyzed root auxin levels in grafted plants. Root stock expressing the reporter gene *GUS* driven by the auxin-responsive *DR5* promoter was grafted with different scions. Compared with wild-type scion, *hy5* mutant scion led to the decreased auxin level at the root tip (Fig. 1B). When the auxin efflux carrier *PIN-FORMED 2* (*PIN2*) or *PIN3* was mutated, root hair development was repressed. However, root hair development could be regained if the *pin2* or *pin3* root stock was grafted with wild-type or *hy5*/*HY5OX* (overexpressing *HY5 in hy5 mutant*) scion, but not *hy5* scion. The results support that HY5 regulates root hair development through the modulation of auxin signaling, and the expression of *HY5* in the shoot can achieve such a regulation in the root.

The authors also addressed the involvement of HY5 in light signaling. In Arabidopsis seedling, light induces IAA accumulation. However, in the absence of HY5, the induction was greatly diminished. In wild-type plants, light increases the number of root hairs. However, when *HY5* was mutated, light-induced root hair development was diminished. The above results further support that HY5 mediates auxin-induced root hair development under light.

In Arabidopsis, RRA1/RRA2 (Reduced Residual Arabinose) regulate root hair elongation, and they are predicted as the targets of miR397b. In this study by Gaddam et al., using 5′ RNA ligase-mediated rapid amplification of cDNA ends, the authors showed the cleavage of RRA1/RRA2 transcripts by miR397b. Sequence analysis revealed a light-responsive element in the promoter of miR397b. By electromobility shift and chromatin immunoprecipitation assays, the authors showed that HY5 binds to the light-responsive element in the *RRA1/RRA2* promoter. Under light, mutations in *HY5* led to a reduction of miR397b expression and consequently caused an increase in *RRA1* and *RRA2* expression. In addition to miR397b, expression analyses and electromobility shift assays revealed *RSL2* and *EXPA7* as potential regulatory targets of HY5. *RSL2* and *EXPA7* encode ROOT HAIR DEFFECTIVE SIX-LIKE 2 and EXPANSIN A7 respectively, which are known to be root hair developmental genes.

In this study, the authors uncovered molecular mechanisms by which HY5 produced in shoots regulates light-induced root hair development. They showed that HY5 is involved in the modulation of auxin signaling and the miR397b-RRA1/RRA2 expression module. The shoot-to-root communication gives an example of long-distance signaling, and it will serve as a model for future studies on long-distance signaling events.
